# Parapatric distribution and sexual competition between two tick species, *Amblyomma variegatum* and *A. hebraeum* (Acari, Ixodidae), in Mozambique

**DOI:** 10.1186/s13071-015-1116-7

**Published:** 2015-10-06

**Authors:** L. Bournez, N. Cangi, R. Lancelot, D.R.J Pleydell, F. Stachurski, J. Bouyer, D. Martinez, T. Lefrançois, L. Neves, J. Pradel

**Affiliations:** CIRAD, UMR CMAEE, F-97170 Petit-Bourg, Guadeloupe France; INRA, UMR 1309 CMAEE, F-34398 Montpellier, France; Université des Antilles et de la Guyane, F-97159 Pointe-à-Pitre, Guadeloupe, France; Centro de Biotecnologia- Eduardo Mondlane University, Av. de Moçambique, km 1,5, C.P. 257, Maputo, Mozambique; CIRAD, UMR CMAEE, F-34398 Montpellier, France; Institut Sénégalais de Recherches Agricoles, Laboratoire National d’Elevage et de Recherches Vétérinaires, BP 2057 Dakar – Hann, Senegal; CIRAD, F-97130, Capesterre-Belle-Eau, Guadeloupe, France; Department of Veterinary Tropical Diseases, Faculty of Veterinary Science, University of Pretoria, Private Bag x04, Onderstepoort, 0110 South Africa

**Keywords:** Exclusive competition, Reproductive interference, Communicative interference, Parapatry, Hybrid inviability

## Abstract

**Background:**

*Amblyomma variegatum* and *A. hebraeum* are two ticks of veterinary and human health importance in south-east Africa. In Zimbabwe they occupy parapatric (marginally overlapping and juxtaposed) distributions. Understanding the mechanisms behind this parapatry is essential for predicting the spatio-temporal dynamics of *Amblyomma* spp*.* and the impacts of associated diseases. It has been hypothesized that exclusive competition between these species results from competition at the levels of male signal reception (attraction-aggregation-attachment pheromones) or sexual competition for mates. This hypothesis predicts that the parapatry described in Zimbabwe could also be present in other countries in the region.

**Methods:**

To explore this competitive exclusion hypothesis we conducted field surveys at the two species’ range limits in Mozambique to identify areas of sympatry (overlapping areas) and to study potential interactions (communicative and reproductive interference effects) in those areas. At sympatric sites, hetero-specific mating pairs were collected and inter-specific attractiveness/repellent effects acting at long and short distances were assessed by analyzing species co-occurrences on co-infested herds and co-infested hosts.

**Results:**

Co-occurrences of both species at sampling sites were infrequent and localized in areas where both tick and host densities were low. At sympatric sites, high percentages of individuals of both species shared attachment sites on hosts and inter-specific mating rates were high. Although cross-mating rates were not significantly different for *A. variegatum* and *A. hebraeum* females, attraction towards hetero-specific males was greater for *A. hebraeum* females than for *A. variegatum* females and we observed small asymmetrical repellent effects between males at attachment sites.

**Conclusions:**

Our observations suggest near-symmetrical reproductive interference between *A. variegatum* and *A. hebraeum*, despite between-species differences in the strength of reproductive isolation barriers acting at the aggregation, fixation and partner contact levels. Theoretical models predict that sexual competition coupled with hybrid inviability, greatly reduces the probability of one species becoming established in an otherwise suitable location when the other species is already established. This mechanism can explain why the parapatric boundary in Mozambique has formed within an area of low tick densities and relatively infrequent host-mediated dispersal events.

**Electronic supplementary material:**

The online version of this article (doi:10.1186/s13071-015-1116-7) contains supplementary material, which is available to authorized users.

## Background

*Amblyomma variegatum* and *A. hebraeum* (Acari, Ixodidae) are two tick species of veterinary and public health concern in Africa (and in the Caribbean for *A. variegatum*) [1–3]. They are the cyclical vectors of *Ehrlichia ruminantium*, the causative bacterial agent of heartwater, a fatal disease of ruminants [4]. In addition, *A. variegatum* greatly facilitates, via immunosuppression in its cattle hosts, the development of dermatophilosis, a skin disease caused by *Dermatophilus congolensis* that imposes major economic impacts [5]. Moreover, human pathogens including several species of Rickettsiae and viruses are transmitted by these ticks [1, 3]. Understanding the factors limiting their geographical distributions is a prerequisite for predicting potential changes in tick distributions and epidemiological risk of associated diseases.

Whereas *A. variegatum* is widely distributed in Africa, its southern limit (Mozambique, Zimbabwe and Botswana) corresponds to the northern limit of *A. hebraeum*’s geographical range, which also extends into South Africa and across Swaziland [6]. In Zimbabwe, the two species were allopatric (i.e. with separated and non-abutting distributions) from the 1930s onwards until civil war stopped acaricide-based tick control in 1975 [7]. An extensive field survey conducted 30 years later revealed a parapatric distribution (i.e. abutting and marginally overlapping distributions) with rare co-occurrences in the same locations [8]. Exclusive competition between the two species has been hypothesised as a mechanism explaining the parapatry seen in Zimbabwe [7, 9]. This hypothesis predicts that similar parapatric range limits could also exist in Mozambique and Botswana. However, there is currently insufficient data at the range limits to know whether or not the parapatry seen in Zimbabwe is present in those countries too. In Mozambique, tick presence data arising from occasional sampling between 1940 and 1975 was only recorded at the district level giving rise to a geographical uncertainty of some hundreds of kilometres [10, 11 & Travassos Santos Dias J,(unpublished data)]. These data suggested the contact zone between these two species was located somewhere near the Save river (19^th^-23^rd^ southern parallels): in the east of the country, *A. variegatum* was found south of the river together with *A. hebraeum* (Govuro and Vilankulo districts) whereas in the west it was the only species recorded to the north of the river and was never found south of the river (Additional file 1: Figure S1). Between 2000 and 2010, only a few tick presence data records arose from opportunistic sampling in the western-part of this area (Neves, unpublished data). These data indicated the presence of *A. hebraeum* to the north-west of the Save river at a distance of 150–200 km from the nearest *A. variegatum* records (Additional file 1: Figure S1).

Sharp distribution boundaries between species may be explained either by marked environmental gradients that traverse the limits of each species’ tolerance to abiotic conditions, or by biotic interactions [12, 13]. These mechanisms give rise to “ecological parapatry” and “competitive parapatry” *sensu lato* respectively [13]. The latter can arise from inter-specific competition *sensu stricto* (e.g. [14–17]), differential effects of pathogens or predators on the two species (e.g. [18, 19]) or reproductive interference (e.g. [20]) (i.e. “any kind of inter-specific interaction during the process of mate acquisition that adversely affects the fitness of at least one of the species involved and that is caused by incomplete species recognition” [21]).

Numerous physiological observations suggest that inter-specific communicative and reproductive interference can occur when these species meet. Both species are three-host ticks, i.e. larvae, nymphs and adults must quest for and feed on different hosts while between stages molting occurs in the environment after blood-fed ticks detach from their hosts. Observations made within their respective geographical ranges have shown that the two species share the same host preferences: adults feed preferentially on large ruminants and have similar feeding-site preferences on hosts [22]. In regions of south-east Africa where the annual rainfall pattern is unimodal it is principally during the rainy season (September to April) that adults (of either species) are observed on hosts, larvae are observed at the end of the rainy season and nymphs during the cold season (May to October) [22]. Experimentally, both species survive under large and overlapping ranges of humidity and temperature [23, 24]. They display similar host and mate seeking behaviour: after several days of fixation on their host, adult males (of both species) attract unfed females as well as other host-seeking males by the emission of attraction-aggregation-attachment pheromones (AAAPs) [25]. These AAAPs are composed of several volatile compounds that differently act as (i) long-range attractants facilitating host location and selection, (ii) aggregation stimulants and (iii) attachment stimulants. Some of these compounds are quite similar between the two species and have been observed to induce partial and asymmetric inter-specific attraction, aggregation and attachment responses in males and females in laboratory experiments [26, 27]. Pheromones isolated from extracts of unmated females of the two species have also been found to share common compounds [28]. Although the exact role of these later pheromones is unknown, they might contribute to aspects of the mating process such as short-distance attraction of males or partner recognition. Finally, the coupling of *A. variegatum* females with *A. hebraeum* males (and vice versa) has been observed to result in hybrid inviability in laboratory experiments [9, 29].

These elements indicate many similarities in the trophic preferences, seasonality and communication systems (pheromones) of these two species. Thus, inter-specific communicative interferences might occur between males or/and between females or/and between males and females of the two species: pheromones produced by one species might inhibit or reduce long and short distance attraction and attachment responses of individuals of the other species to their own pheromones through inhibition effects or inefficient signal reception. Such effects might induce competition for hosts (i.e. competition for free attachment sites on host or for signal reception) and lead to spatial segregation of attachment positions between the two species in sympatric areas (i.e. attachment on different hosts or on different attachment sites within the same hosts). By contrast, these pheromones might have inter-specific attractivity effects and might induce strong sexual communication errors (i.e. sexual competition) resulting in inter-specific mating. Although cross-mating has been observed between *A. variegatum* and *A. hebraeum* under experimental conditions [9, 29], its existence and importance in the field is unknown. For example, whether or not sexual competition is avoided or mitigated by spatial segregation of males of the two species at sympatric sites is unknown.

To explore the hypothesis of competitive exclusion between *A. variegatum* and *A. hebraeum* we conducted a field survey at their range limits in Mozambique (1) to analyse their spatial distribution and (2) to assess the strength of inter-specific communicative and reproductive interference effects at co-infested sites.

## Methods

### Distributions of *A. variegatum* and *A. hebraeum* in the Mozambican contact zone

**Fig. 1:**
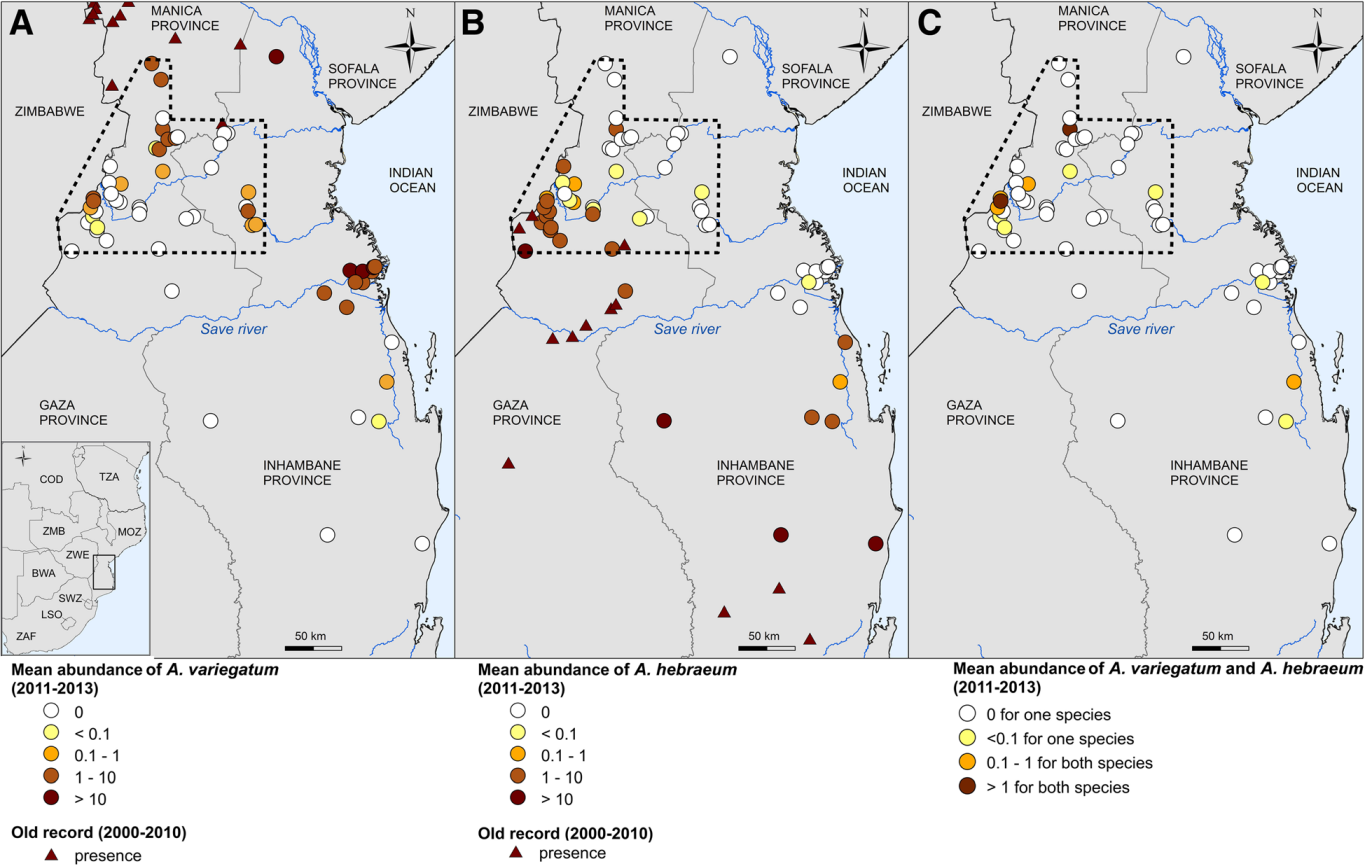
Mean abundance of *Amblyomma* ticks in the Mozambican contact zone, **a** *A. variegatum*, **b** *A. hebraeum* and (**c**) co-occurrences. Dotted lines: limits of quasi-exhaustive sampling of sites with cattle. BWA = Botswana, COD = Democratic Republic of Congo, TZA = United Republic of Tanzania, LSO = Lesotho, MOZ = Mozambique, SWZ = Swaziland, ZMB = Zambia, ZWE = Zimbabwe, ZAF = South Africa

During 2012–2013, we conducted field surveys along north–south and east–west transects crossing the known range limits of both species (Additional file 1: Figure S1) in Inhambane, Manica, and Sofala provinces (19^th^-23^rd^ southern parallels, Fig. 1) in two consecutive rainy seasons (peak adult *Amblyomma* activity period). In February 20112, we sampled cattle at sites spaced 30–50 km apart to identify the location and extent of the contact zone. In February 2013, we sampled cattle from sites spaced 5–10 km apart in the south of Manica and Sofala provinces, we refer to this area as the “area of quasi-exhaustive sampling” since our survey visited a large majority of map pixels (3 × 3 km) with farms in that area. Study sites included communal dip-tanks and corridors used for acaricide treatment by farmers, plus some farms with no access to such facilities. When possible, a minimum of 50 animals within 3 km (approximate grazing range) of sampling locations, or (when less) all animals present on farms, were examined for tick presence and species identification. When feasible, 10 of these animals were laid down for detailed examination and the remaining cattle were examined in the corridor. During clinical examinations we counted the total number of adults of each *Amblyomma* species on each animal. Four herd infestation levels were defined according to mean abundance of ticks (i.e. number of *Amblyomma* adults/number of animals examined): <0.1, [0.1 – 1), [1–10) and ≥ 10 adults *Amblyomma* per animal^1^. We only included in the analysis animals that were treated with the acaricide product Amitraz eight days or more prior to the visitor those treated with pyrethroids 15 days or more prior the visit. These products were the only ones used on sampled farms. Eight days and fifteen days represent the mean duration of residual effects on hosts of Amitraz and pyrethroids respectively and the short-time needed for attraction and attachment of ticks on hosts. *Amblyomma* data were mapped to enable visualisation of the spatial distributions of the two species and their range limits.

In order to estimate the probability to detect ticks on cattle in a given area, cattle population data, encompassing census information, cattle movements and herd management practices including tick control, were collected through interviews with farmers and local veterinary service staff. At each location the probability to detect ticks in a herd, given the sample size, an assumed prevalence of 20 % and an assumed probability of tick detection on an infested host (“sensitivity”) of 1 for inspections on laid-down animals and 0.6 for corridor inspections, was deemed “high” when it was estimated to be greater than 0.7 (Additional file 1: Method 1). These probabilities were also mapped.

Since the abundance of *A. variegatum* and *A. hebraeum* in a given area is conditionally related to the presence of large ruminants, we collected qualitative data on the presence of large wild ruminants and cattle. Cattle densities were mapped at a 10-km resolution to help identify potential tick infestation areas in the field and identify areas where low cattle host densities might amplify stochastic effects in the stability of *Amblyomma* populations. For this, cattle census data were available in the districts of Govuro, Chibababva, Sussudenga, Mossurize and Mabote (Additional file 1: Figure S2). For the other districts,we used the FAO’s modelled data “Gridded Livestock of the World v2.01” [30]. All maps were produced using ArcMap v10 [ESRI, Redlands, California, USA].

### Inter-specific communicative interference between *A. variegatum* and *A. hebraeum*

#### *Attachment site preferences of* A. variegatum *and* A. hebraeum *with and without co-infestation*

To assess potential differences in the attachment site preferences of the two species on co-infested animals, we measured overlap in adult attachment site preferences of each species using Schoener’s D index [31]: D_H_ = 1-1/2 ∑_*i*_ |*p*_Av,*i*_ –*p*_Ah,*i*_|, where *p*_Av,*i*_ and *p*_Ah,*i*_ are the proportion of *A. variegatum* and *A. hebraeum* attached at site *i*on all co-infested animals. Attachment sites were divided into perinea-thigh region, inguinal region, axillary region, belly, head, legs, tail and dewlap.

To assess whether or not ticks modified their attachment site preferences in the presence of the other species, Schoener’s D index was used to compare attachment site preferences of *A. variegatum* (or *A. hebraeum*) on animals infested by a single tick species with those observed on co-infested animals: D_C_ = 1-1/2 ∑_*i*_ |*p*_O,*i*_ –*p*_T,*i*_|, where *p*_O,*i*_ and *p*_T,*i*_ are the proportions of *A. variegatum* (or *A. hebraeum*) attached at site *i* for all animals infested by one (O) or two (T) *Amblyomma* species respectively. Both D_H_ and D_C_ were calculated separately for male and female ticks and for three cattle infestation levels (*n* < 30, 30 ≤ *n* < 70, *n* ≥ 70; with *n* the total number of ticks) using data from laid-down animals only.

#### Co-occurrence patterns at animal, attachment site and cluster levels

To assess the existence of between-species attraction or repulsion we analysed the spatial distribution (i.e. segregated vs. aggregated) of ticks in sympatric sites at the level of: host animal, attachment sites and cluster (a group of ticks aggregated within a 5 cm radius). These levels represent long (<4 m, i.e. average distance of attractive effects toward host-seeking ticks [32, 33]), medium (<30 cm) and short (<5 cm) range effects of pheromones respectively. The number of males and females per host, attachment site and cluster were counted on laid-down animals at sites where abundance of either species was superior to 0.1 adult ticks/host. We compared observed co-occurrences of individuals from the same or different species and/or sex to those generated by 5000 random permutations of data using the checkerboard score (C-score) [34] calculated via the function oecosimu from the R package vegan 2.2-2 [35].

### Reproductive interference between *A. variegatum* and *A. hebraeum* in the field

Reproductive interference was assessed on naturally-infested cattle via the number of females mating with con- or hetero-specific males. Deviation from random mating was estimated at co-infested sites via the pair total index (PTI) and pair sexual isolation index (PSI) [36] with the assumption of random mating of all individuals (PTI) or among individuals that actually mated (PSI). Sexual isolation was estimated via the joint isolation index (I_PSI_) summarizing the difference in overall proportions of con- and hetero-specific pairs [36]. It ranges from −1 (fully hetero-specific mating) to 1 (complete isolation). We assessed asymmetry in hetero-specific mating by the index IA_PSI_, the PSI ratio of hetero-specific combinations [37]. Standard errors (se) were estimated using 10,000 bootstrap replicates of the original data. Two-tail probabilities of obtaining estimates different from 0 for I_PSI_ and different from 1 for PSI, PTI and IA_PSI_ quantified deviations from random expectation (Jmating software [37] version 1.0.8).

Proportions of con-specific mating pairs were analysed using a beta-binomial logistic regression. The response variable was the frequency of con-specific couplings among all mating pairs. Fixed effects included (i) the mated-female species, (ii) the proportions of con-specific males and females centred on 0.5 (i.e., proportion - 0.5) and (iii) the interaction between these two proportions. Centring was used to facilitate the interpretation of model intercept and to decrease the correlation between fixed-effect coefficients. Over-dispersion is common in ecological data and not accounting for it can lead to spurious significance levels in statistical tests [38]. Here, over-dispersion with respect to the binomial distribution was modelled via a within-cattle correlation coefficient *r*. The statistical significance of these effects was quantified using likelihood ratio tests (LRT).

We examined the morphology of 30 ticks (or all when fewer were found) per species per study site under a stereo-microscope to search for phenotypical patterns that differed from those known for *A. variegatum* or *A. hebraeum* and may therefore represent hypothetical co-dominant or incomplete dominant hybridization. Morphological identification of *A. hebraeum* and *A. variegatum* was based on descriptions and observations documented in identifications keys [6, 39–42] of the following morphological criteria: colouration of the festoons, presence of median lateral areas of enamel, convexity of the eyes and nature of the colouration of scutal ornamentation.

Unless stated otherwise, all statistical analyses were performed with R version 3.1.2 [43].

### Ethical approval

All the field work was implemented according to survey protocols approved by the Scientific Board of the Veterinary Faculty of the Eduardo Mondlane University, Maputo, Mozambique. The study permission was obtained from the Mozambican Livestock National Directorate, the Inhambane’s, Sofala’s and Manica’s Livestock Provincial Directorate, from community leaders and from the farmers.

## Results

### Distributions of *A. variegatum* and *A. hebraeum* in the Mozambican contact zone

We sampled 59 sites in the Mozambican contact zone. The sampling effort was considered sufficient to enable high likelihood of species detection at 92 % (51/59) of sites (Additional file 1: Figure S3). We found *Amblyomma* ticks at 49 sites (83 %): 18 (31 %) with *A. variegatum* only, 19 (32 %) with *A. hebraeum* only and 12 (20 %) with both species (Fig. 1, Additional file 1: Table S1). Of these 12 sites: mean abundances of both species was superior to 1 tick/animal at just 2 sites (Fig. 1c, Additional file 1: Table S1), with *A. variegatum* predominant at one site and with approximately equivalent abundance of the two species at the other; mean abundance of both species was between 0.1 and 1 ticks/animal at 3 sites; one species was predominant with few individuals (<0.1 ticks/animal and <5 observed ticks in total) of the other species at 7 sites (Additional file 1: Table S1).

**Fig. 2:**
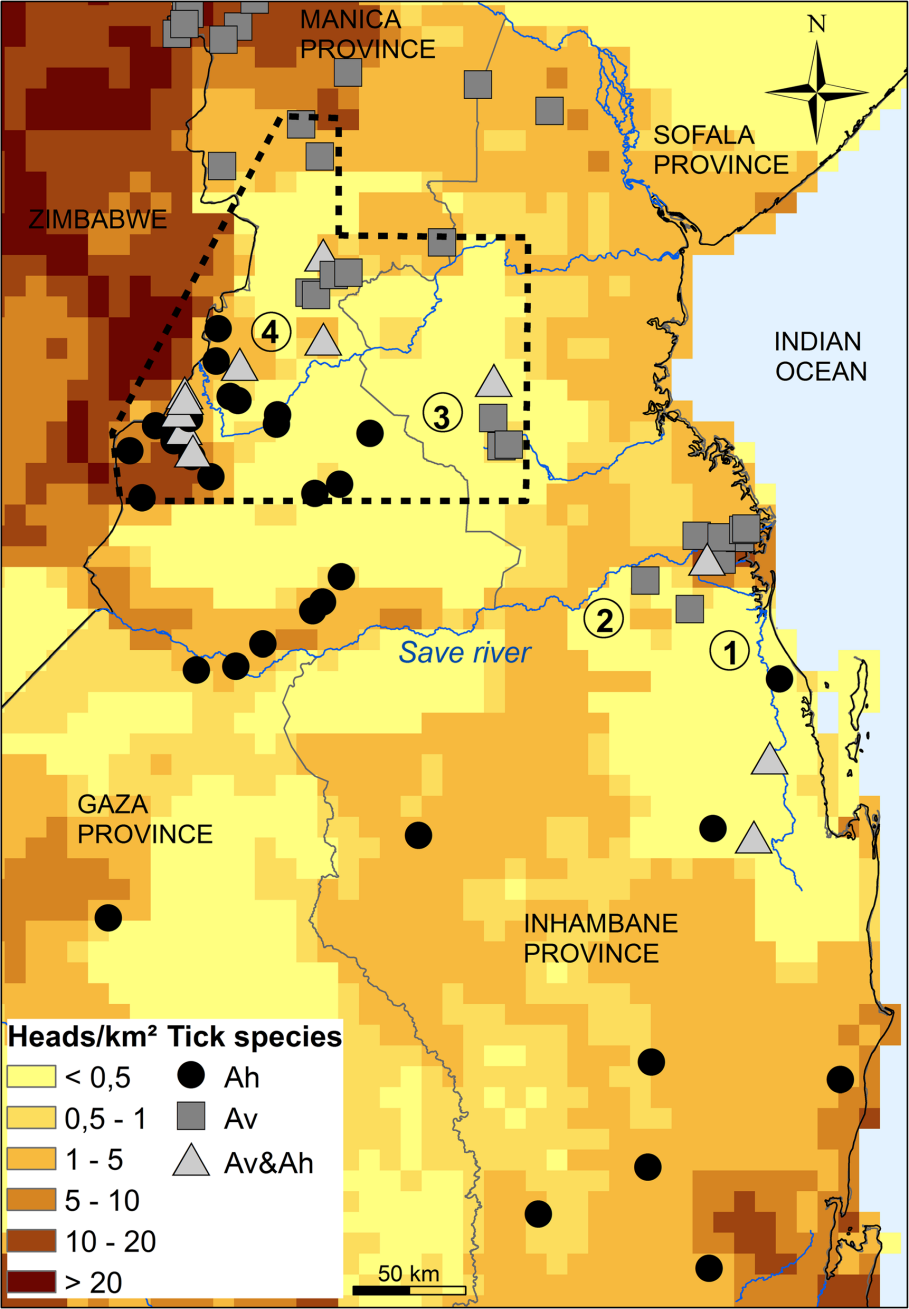
Cattle densities and *Amblyomma* distribution at the Mozambican contact zone. Characters designed areas of contact/transition between populations of *A. variegatum* (Av) and *A. hebraeum* (Ah). Dotted lines: limits of quasi-exhaustive sampling of sites with cattle

In this area, cattle densities were heterogeneous: absence or very low densities (<0.5 heads/km^2^) of cattle was common over much of this region, otherwise cattle densities ranged between 0.5 and 25 heads/km^2^ (Fig. 2). Outside protected areas (national parks, nature reserves) and hunting reserves, densities of wild animals, especially for large ruminants, were extremely low.

Areas of contact or transition between *A. variegatum* and *A. hebraeum* populations were all characterised by very low cattle densities (<0.5 heads/km^2^) and mean tick infestations inferior to 10 ticks/animal. The observed zone within the quasi-exhaustive sampling area (dotted lines in Fig. 1) in which *A. variegatum* and *A. hebraeum* distributions overlapped was approximately 80–90 km wide.

### Inter-specific communicative interference between *A. variegatum* and *A. hebraeum*

#### *Attachment site preferences of* A. variegatum *and* A. hebraeum *with and without co-infestation*

The great majority (>90 %) of males and females of both tick species was attached to the perineal, inguinal and axillae regions of host animals at all study sites. At sympatric sites, the preferred attachment sites of the two species were highly similar on co-infested animals (Schoener’s D_H_ = 0.86 for hetero-specific males, D_H_ = 0.84 for hetero-specific females) (Additional file 1: Tables S2 and S3). However, attachment site preferences of *A. variegatum* on cattle hosting/not hosting *A. hebraeum* were less similar (Schoener’s D_C_ = 0.75 for males, Dc = 0.74 for females) than attachment site preferences of *A. hebraeum* on cattle hosting/not hosting *A. variegatum* (D_C_ = 0.91 for males, Dc = 0.87 for females). Whereas *A. variegatum* attached most frequently in the inguinal region when host animals were not infested by *A. hebraeum*, on co-infested animals *A. variegatum* attached preferentially in the axillae region where *A. hebraeum* was less abundant than in the inguinal region.

#### Co-occurrence patterns at animal, attachment site and cluster levels

At the 5 sites where both species were observed with an abundance superior to 0.1 ticks/animal (Additional file 1: Table S1), 59 % (36/61) of cattle were found to be co-infested by males of both species and 34 (21/61) and 7 % (4/61) respectively were infested only by males of either *A. variegatum* or *A. hebraeum*. The great majority (>80 %) of males and females of both species was attached on co-infested animals (Table 1).

**Table 1:** Within- and between-host co-occurrence of *Amblyomma variegatum* and *A. hebraeum*. Percentage of males and females of *A. variegatum* (Av) and *A. hebraeum* (Ah) attached on/within the same animals, attachment sites or clusters as *A. hebraeum* and *A. variegatum* males

	Av males		Ah males		Av females		Ah females	
	*n*	% attached in presence of Ah males	*n*	% attached in presence of Av males	*n*	% attached in presence of Ah males	*n*	% attached in presence of Av males
Animals	275	80.0	133	96.2	100	81.0	43	100.0
Attachment sites	203	80.8	119	96.6	75	74.7	39	84.6
Clusters	203	52.0	119	74.7	75	49.0	39	71.0

On co-infested animals (*n* = 32 animals with tick cluster data), 55.5 % (*n* = 72) of attachment sites and 35.5 % (*n* = 121) of clusters were infested by males of both species and 33.3 and 41.3 % respectively were infested by males of *A. variegatum* but not by males of *A. hebraeum*. The majority of ticks (>50 %) of the two species were attached close to each other within the same attachment site or the same cluster (Table 1). However, males and females of *A. variegatum* attached less frequently in common attachment sites and clusters than those of *A. hebraeum* (Table 1). Animal tick infestation was low: the median [25^th^ percentile; 75^th^ percentile] number of males and females per co-infested animal was 4 [2.25; 9] and 2 [1; 3] for *A. variegatum* and 2 [1.25; 4] and 1 [0; 3] for *A. hebraeum* respectively.

**Fig. 3:**
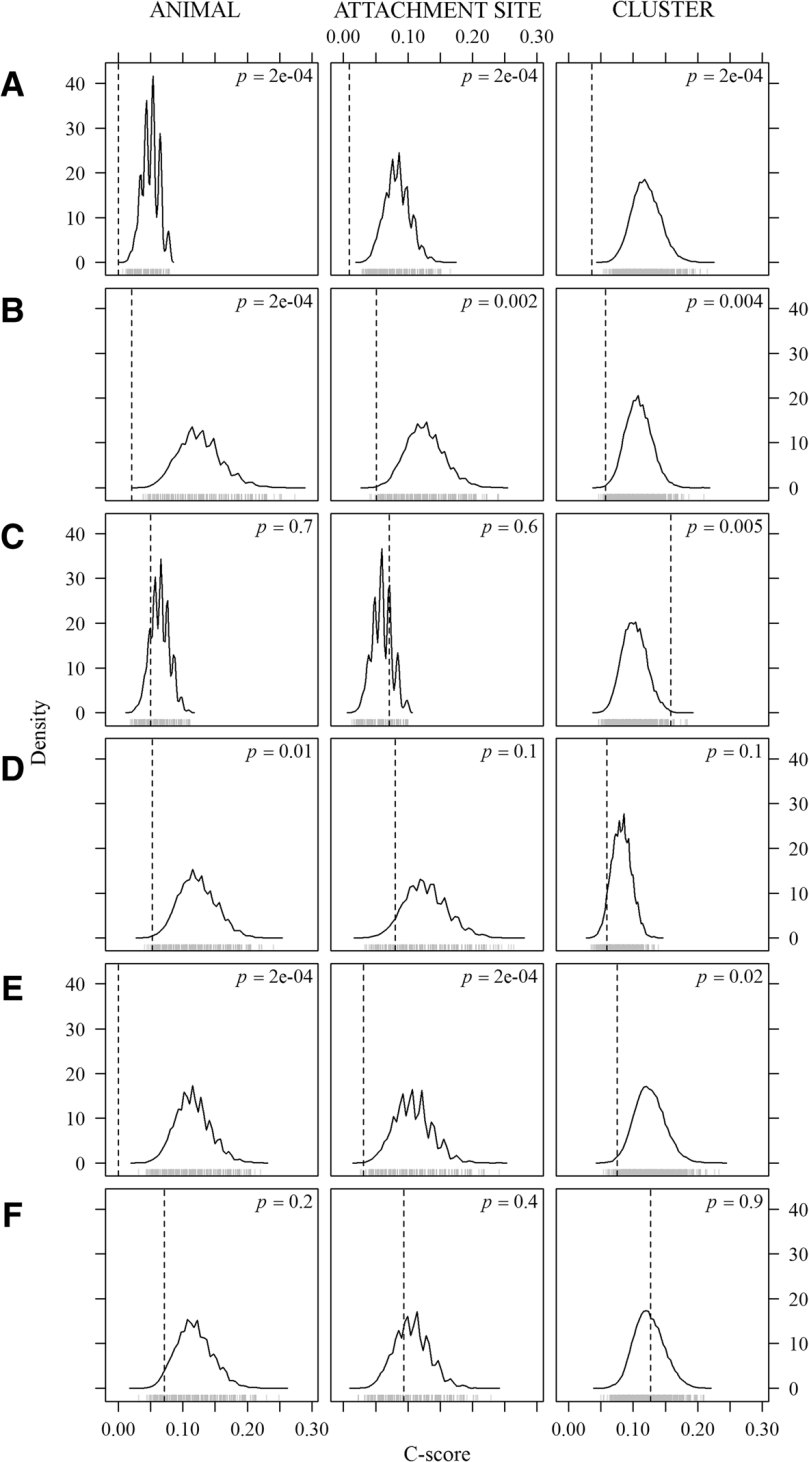
C-scores of tick pairs according to species and sex at host, attachment site and cluster level (dotted line) compared to frequency distributions (solid line) generated from 10,000 Monte Carlo simulations using equivalent frequencies for each group and independent distributions (null model). Tested pairs are: **a** *A. variegatum* males vs females, **b** *A. hebraeum* males vs females, **c** *A. variegatum* males vs *A. hebraeum* males, **d** *A. variegatum* females vs *A. hebraeum* females, **e** *A. variegatum* males vs *A. hebraeum* females, **f** *A. hebraeum* males vs *A. variegatum* females

Whereas males of the two species were independently distributed at the host and attachment site levels (C-score = 0.05*, p* = 0.57 and C-score = 0.07*, p* = 0.67), they were more segregated than expected by chance at the cluster level (C-score = 0.16, *p* = 0.005, Fig. 3) indicating that only short range aggregation was affected by potential competitive effects between hetero-specific males. *Amblyomma variegatum* males formed hetero-specific clusters less frequently than expected by chance, while the situation was reversed for *A. hebraeum* males (*χ*^2^ = 13.8, *p* < 10^−3^). The presence of *A. variegatum* males significantly increased the probability of the presence of *A. hebraeum* females at the animal (C-score = 0, *p* <0.001), attachment site (C-score = 0.03, *p* < 0.001) and cluster levels (C-score = 0.07, *p* = 0.017); however, the reciprocal relation was not true (C-score = 0.07, *p* = 0.22; C-score = 0.009, *p* = 0.34; C-score = 0.13, *p* = 0.88 respectively). As males of both species were independently distributed at host and attachment site levels, these effects cannot only be attributed to the presence of *A. hebraeum* males and suggest that *A. variegatum* males may also induce long-distance attraction effects to *A. hebraeum* females. Given the negative association between the distributions of males of both species at the cluster level, females of both species appeared to be locally attracted by groups of hetero-specific males, with a stronger effect observed among *A. hebraeum* females. Females of the two species were aggregated at the host level (C-score = 0.05, *p* = 0.036) but distributed independently at the attachment site (C-score = 0.08, *p* = 0.67) and cluster levels (C-score = 0.06, *p* = 0.12).

### Reproductive interference between *A. variegatum* and *A. hebraeum* in the field

Cross-mating was observed at the two sites where abundance of both species was superior to 1 tick/animal (sites # 12 and 55 in Additional file 1: Table S1). At one of these sites *A. variegatum* was more abundant among observed males, whereas at the other site males of the two species were approximately equi-abundant. At the latter site, 15.5 (9/58) of *A. variegatum* females and 12.5 % (4/32) of *A. hebraeum* females mated with hetero-specific males, whereas 69 % (40/58) of *A. variegatum* females and 62.5 % (20/32) of *A. hebraeum* females mated with con-specific males and 15.5 % (9/58) of *A. variegatum* females and 25 % (8/32) of *A. hebraeum* females were attached single. *Amblyomma variegatum* and *A. hebraeum* showed substantial but incomplete sexual isolation (I_PSI_ = 0.65, se = 0.09, *p* < 10^−3^; PSI and PTI values of hetero-specific pairs between *A. variegatum* males and *A. hebraeum* females were 0.27 and 0.28 respectively, and PSI and PTI of hetero-specific pairs between *A. hebraeum* males and *A. variegatum* females were 0.46 and 0.42 respectively, all PSI and PTI values were significantly different from 1, *p* < 0.05). We found no evidence of asymmetry in hetero-specific mating (IA_PSI_ = 1.99, se = 1.16, *p* = 0.2). At the former site (where *A. variegatum* was dominant), 15 (4/26) of *A. variegatum* females and 100 % (4/4) of *A. hebraeum* females mated with hetero-specific males; conversely, 77 % (20/26) of *A. variegatum* females mated with con-specific males. All the ticks examined under a stereo-microscope were identified as being either *A. variegatum* or *A. hebraeum* and no intermediate forms observed.

**Fig. 4:**
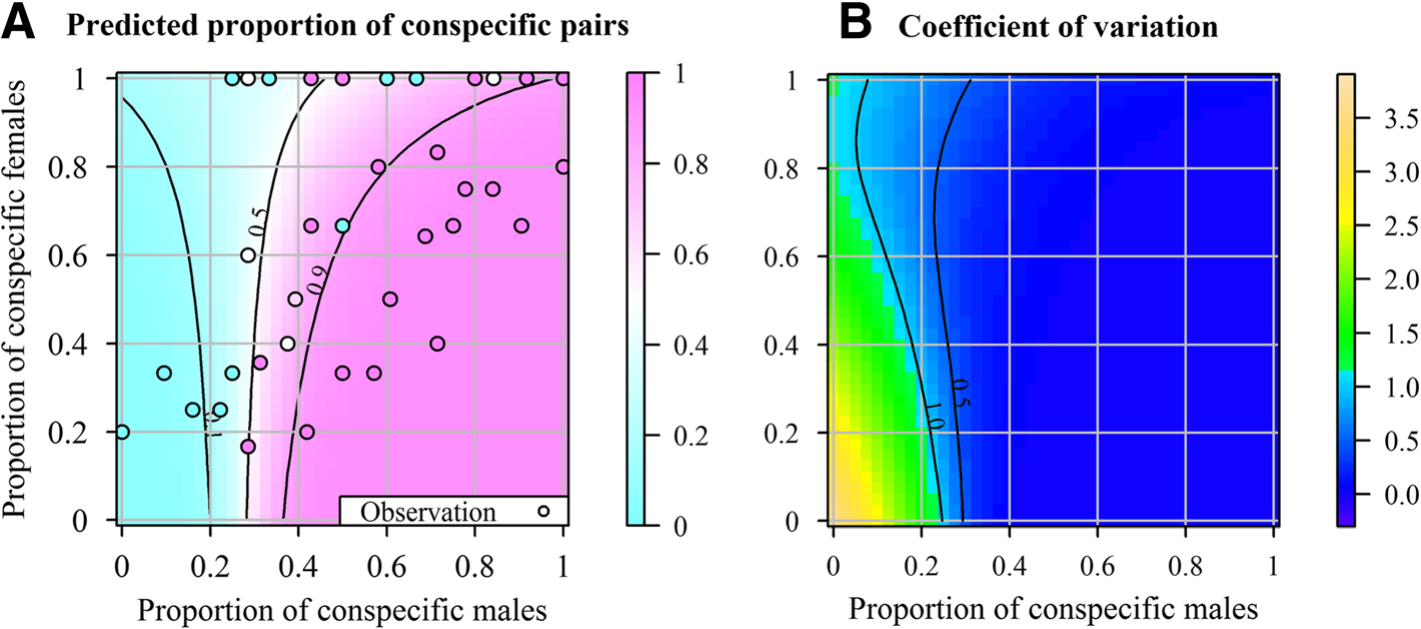
Predicted (colour) and observed (circle) distribution of con-specific mating ticks according to the proportion of con-specific males and females. **a** mean proportion and **b** coefficient of variation predicted by a beta-binomial logistic regression model. Contour lines indicate the predicted probabilities to observe proportions of con-specific mating ticks of 0.1, 0.5, and 0.9

The beta-binomial logistic regression model of the frequency of con-specific couplings among mating pairs presented a high within-cattle correlation coefficient (*r* = 0.37, Pr(> |*r*|) = 0.017). The effect of female tick species was small and not significant (Additional file 1: Table S4), in agreement with the IA_PSI_ index. The effects of both proportions of con-specific males and females were significant (Additional file 1: Table S4) and the interaction between the proportions of con-specific males and females was strong and negative (LRT, *χ*^2^ = 10.4, df = 1, *p* = 0.001). Model predictions provided evidence for a preference of females for males of the same species: 67 % of the surface of Fig. 4a (magenta colour) corresponds to a majority of mating pairs being con-specific. The generally low coefficient of variation indicates that the accuracy of these predictions can be expected to be good, except when the proportions of con-specific males and females were both low (Fig. 4b). Large predicted proportions of con-specific mating pairs were obtained when the proportion of con-specific males was >30 %, except when high proportions of con-specific females (>70 %) were encountered (Fig. 4a). Even in this case, proportions of con-specific males > 50 % resulted in high predicted proportions of con-specific mating pairs. The effect of an increase in the proportion of con-specific females on the predicted proportion of con-specific pairs was different when the proportion of con-specific males was inferior (vs. superior) to 25 %: below this threshold, predicted proportions of con-specific pairs increased slightly when the proportion of con-specific females increased; inversely, above this threshold predicted proportions of con-specific pairs decreased faster as the proportion of con-specific females increased.

## Discussion

Our observations confirm the existence of a parapatric boundary between the geographic distributions of *A. variegatum* and *A. hebraeum* populations in Mozambique. The adopted sampling design was based on the well documented phenology of these species – when these species are present in a given area, adults are mainly observed on cattle throughout the rainy season and it would be extremely rare not to observe *Amblyomma* during this period unless abundances were very low. Even when observations were made as late as February, a period that usually corresponds to the decreasing phase of adult infestation curves, adult infestation levels are still expected to be sufficiently high to have a large probability to observe *Amblyomma* ticks when present at moderate to large numbers. Indeed, we did observe both species at several sites during this period. To facilitate interpretation of observed absences we calculated the conditional probability to detect tick presence given our sampling at each site, an assumed 20 % of tick prevalence in the herd (which is low for these species) and an assumed sensitivity of tick detection on an infested host of 1 for inspections on laid-down animal and of 0.6 for corridor inspections. Based on our collective experiences on the field these figures are the minimum expected for scenarios of low levels of tick infestation such as 1–5 ticks per animal (Additional file 1: Method 1). These conditional probabilities were greater than 0.7 at the majority of sampled sites (median 0.98, range 0.41- 0.99). As such, the observed absence of one species at any given site most likely reflects either a true absence or a very low abundance.

In this area, we found coexistence of both species at relatively few sites. At most of the co-infested sites, densities of one or both species were low (<1 tick/animal) and often less than 5 individuals of the sub-dominant species were collected in total. Outside the quasi-exhaustive sampling area, e.g. in Inhambane province, we were able to visit only a limited number of farms due to organisational constraints. This impedes accurate estimation of the number of co-infested sites in an area 200 km wide. In the quasi-exhaustive sampling area where we visited a large majority of map pixels with cattle farms, co-infested sites were located in a zone of 80–100 km in width. Historical tick records from 2009 and 2010 located in Manica province, northern and southern from this area, indicated the presence of a single *Amblyomma* species as well and hence may be perceived as additional hints to indicate that sympatric areas were limited to the study area. In most sites that were not sampled in this area, *Amblyomma* abundance can be expected to be very low or zero due to the absence or a very low abundance of large ruminants (cattle or large wild ruminants). Indeed, although under such conditions medium-size animals might serve as alternative hosts for adults of *A. variegatum* and *A. hebraeum* as evidenced experimentally, there is no evidence that under natural conditions these species can persist in areas where there are no large ruminants. The number of adult ticks that feed successfully on small animals is much lower than on large ruminants [44–53] and this appears to impede the tick’s reproductive performance [54, 55]. Therefore, although it is possible that some co-infested sites might have escaped detection, especially where abundances of one species were low, our results do indicate that these species rarely co-exist at high or moderately high abundance levels. A similar pattern was observed in the contact zone in Zimbabwe during the last survey conducted in 1996 [8]. This pattern is consistent with a dynamical system in which sympatry, when it arises, is highly transient. More generally, *Amblyomma* densities observed in this area were low (<10 ticks*/*animal), compared to densities observed in the rest of our study area (>20 ticks/animal) or to previous reports of *Amblyomma* densities from other areas [56–61]. Our data do not permit accurate tick density estimates at each site, however, the between-site differences detected here during the peak adult tick activity season are sufficiently large to be highly informative regarding the location of the parapatric boundary in Mozambique within an area of low tick abundance. Further, heterogeneity in cattle distribution combined with very low densities of large wild ruminants within the contact zone probably renders tick densities highly heterogeneous across this area. As discussed previously, it can be expected that in areas with no or few large ruminants, *Amblyomma* abundance is either very low or zero.

One hypothesis to explain the parapatric distribution between *A. variegatum* and *A. hebraeum* is that some form of inter-specific competition *sensu lato* impedes the spread of either species across the contact zone. Our field observations suggest competition for sexual partners, together with the absence of strong repellent effects between species, could provide a mechanism to generate this parapatry. We observed that adults of both species preferred the same three main attachment sites on hosts (axillae, inguinal and perineal region), whether or not the other species was present. Moreover, they frequently attached to the same hosts and in the same clusters. High cross-mating rates (12.5 and 15.5 % respectively for the females of *A. hebraeum* and *A. variegatum*) were observed in the field when the two species were equally abundant. Thus, females often fail to correctly identify their sexual partners and reproductive isolation at pre-mating barriers is only partial. This phenomenon can be attributed to an inability to fully distinguish between species via the pheromones emitted for long or short distance attraction, attachment and mating.

Cross-mating rates did not differ significantly with the species of the female, thus asymmetry in reproductive interference effects was undetectably small. By contrast, asymmetry was detected in the mating preferences of females: we found the attraction-attachment effects of hetero-specific males to be stronger for *A. hebraeum* females than for *A. variegatum* females. This is consistent with experimental results of Norval et al. [26, 27] who used extracts of tick pheromones. Therefore, other mechanisms of recognition are probably at play downstream in the reproductive cycle that reduce this asymmetry. Rechav et al. [9] made similar observations: although a large number of *A. hebraeum* females attached close to *A. variegatum* males, only a small proportion actually formed hetero-specific pairs. The opposite was observed with *A. variegatum* females, which were less attracted by males of *A. hebraeum* but of which a significant proportion formed hetero-specific mating pairs.

Such “mating errors” can have a huge impact on reproductive success, the most extreme scenario being when the attraction of females towards hetero-specific males results in a complete failure to mate with con-specific males – such phenomenon greatly increase the local extinction probability of the least frequent species. We observed no major differences between the two species in the percentage of single females attached to a host when male abundances were equal, suggesting symmetry or very small asymmetry of competitive effects at that level, although in general symmetry or small asymmetry in reproductive interference effects is rare among taxa [21]. In addition, communicative interference between males could also influence the reproductive fitness of the two species. Indeed, within clusters, males of the two species were more segregated than expected under randomisation, suggesting a local and partial repellent effect between them. The repellent effect of *A. hebraeum* males towards *A. variegatum* males was more pronounced, as observed by Norval et al. [26, 27]. This may also explain the slight difference observed in *A. variegatum*’s attachment site preferences in the presence or absence of *A. hebraeum*. However, further experiments are needed to more accurately estimate the size and degree of asymmetry in deleterious effects (on each species reproductive success) arising from communicative interference between males, mismating and hybridization.

Results of previous hetero-specific-cross experiments suggest that cross-mating between *A. variegatum* and *A. hebraeum* is most likely to generate inviable eggs [9, 29]. This implies that, if hybrids are generated, then this occurs at undetectably low frequencies. We did not observe ticks with morphologically intermediate forms that may represent hypothetical co-dominant or incomplete dominant hybridization at sympatric sites. When cross mating results in inviable offspring, a frequency-dependent mechanism, called a Satyr effect or satyrisation [62], is produced that can lead to exclusive competition and parapatry between species regardless of whether or not the exclusion generated by satyrisation is enhanced by exploitative competition (i.e. competition for resources or apparent competition through shared predators or pathogens) [62–65]. Satyrisation is predicted to decrease reproductive fitness as the relative proportion of hetero-specific individuals increases. In the Mozambican contact zone, the frequencies of hetero-specific males and (to a lesser extent) hetero-specific females both appear to influence reproductive fitness. The beta-binomial regression model of con-specific pairs at the animal level predicted that the proportion of con-specific mating pairs increases with the proportion of con-specific males, the degree of increase depending on the proportion of con-specific females. Low con-specific mating rates were predicted when the proportion of con-specific males was inferior to 0.3. Above this threshold, the proportion of con-specific pairs was generally high (>0.5) but decreased slightly as the proportion of con-specific females increased. This is coherent with our observation of high hetero-specific mating rates of *A. variegatum* females (15 %) at the sympatric site where this species was dominant. Such influences of the relative frequencies of con-specific and hetero-specific females on reproductive fitness were unexpected for this species given that males can remain on the host for several months and mate with several females and are generally more numerous than females. This may reflect that con-specific males were a limited resource for females at the animal level, which might be related to (i) the low density of ticks of any species in the study area and consequently, the low number of male ticks on infested cattle (most animals were infested by <5 male ticks), (ii) frequent acaricide treatments of cattle (usually every 15 days) reducing the number of male ticks that have been attached for long enough (at least 5–7 days) to emit AAAP pheromones and attract females; (iii) a heterogeneous proportion of the two species on animals or in the environment. Thus, sexual competition appears to decrease the population growth rate of both dominant and sub-dominant species at sympatric sites increasing the sensitivity of low populations to stochastic events. However, such effects are generally not considered in mathematical studies [62–64].

The positive frequency-dependent effect and near-symmetry of reproductive interference can be expected to induce local extinction to the least abundant species at sympatric sites [62–64]. Spatially explicit theoretical sexual competition models [62, 65, 66] predict that the dominant species at a given location at some time *t* is determined by (i) “initial conditions” i.e. the relative frequency of each species at some previous time *t*_0_ at each location within the landscape, (ii) the fitness of each species given the abiotic conditions and any modifications to fitness caused by inter-specific competitive effects such as the satyrisation effect, and (iii) dispersion rates, which can be spatially heterogeneous. These models predict that the invasion of a fitter species into an area where another species is established will be unsuccessful unless the number of invading individuals exceeds a certain threshold. Otherwise, frequency dependent competition effects prevent a successful invasion and the newly introduced species typically disappears in just a few generations in the absence of continuous immigration. The influences of “initial” abundances of a resident species on the final outcome of competition with an invading species are known as “priority effects”. These models also predict that when populations are initially allopatric and dispersal rates are high parapatric boundaries tend to form close to isoclines of environmental gradients that delimit equivalence in density independent fitness. But, lower dispersal rates can strengthen priority effects, accentuate the impacts of stochastic events and generate greater variation in the exact locations of parapatric boundaries.

Given the high bidirectional hetero-specific mating rates observed between *A. variegatum* and *A. hebraeum* it can be expected that strong priority effects could be generated by reproductive interference and that this process could strongly influence the limits of their geographical distributions, particularly when the spread of one species into the range of another is limited by low dispersal rates. This might occur at the contact zone in Mozambique. Tick dispersal is dependent upon host movement and the flow of cattle and wild ruminant host animals across this contact zone is thought to be low (national veterinary services, pers. comm.). Low tick and host densities as well as the patchy distribution of hosts observed at the contact zone may result in relatively infrequent tick dispersal events across the area. Some well-established populations of *A. variegatum* and *A. hebraeum* were found to be separated by “no-cattle lands” as wide as 40–60 km – such areas can be expected to function as dispersal barriers to ticks. However, even if tick dispersal events are infrequent, they are not completely negligible as suggested by the presence of a small number of individuals of the non-dominant species at seven out of the 59 investigated sites and reported movements of cattle and small ruminants across the area (national veterinary services, pers. comm.). Thus, it is unlikely that infrequent dispersion alone provides a sufficient mechanism to prevent range expansion of both species. However, priority effects generated by sexual competition do appear to explain why the current location of the Mozambican parapatric boundary falls within a zone of apparently low tick densities and infrequent dispersal. Low densities, stochastic events and priority effects suggest that sites of sympatry (and the extent of the overlapping area) are likely to shift stochastically at relatively high frequency, although the dominant species at each site would change relatively infrequently. Existence of competitive exclusion between *A. variegatum* and *A. hebraeum* and how it interacts with abiotic factors (i.e. the relative importance of priority effects) can be explored further by studying habitat suitability for both species, studying tick dispersal across the area and by performing periodic follow up surveys at sites in and around the contact zone over a number of years. Historical tick records are insufficient to be informative about this hypothesis since these data are insufficient in quantity and quality to determine whether or not the location of parapatric boundaries have shifted with time or even which species were historically present in the area. However, it is worth noting that despite massive changes in host populations in the area between 1950 to 2014 (e.g. massive reductions of large ruminant populations (both cattle and wild large ruminants) during the civil war (1975–1992), followed by progressive reintroduction of cattle in traditional farming areas, especially from Zimbabwe), the parapatric boundaries as observed now appear to be located within the same districts as suggested by records from the 1950’s. The extent of which the location of the parapatric is determined by environmental factors remains unknown (but see [67] for Zimbabwe’s parapatric boundaries) and warrants further study. However, this uncertainty does not negate our main conclusion, namely that satyrisation is a key mechanism that can inhibit coexistence between these two species of tick and can inhibit the invasion of one species into an area where the other species is already abundant even when the resident species is less fit than the invader given the local environmental conditions.

When two closely related species are in regular contact, pre-mating barriers can evolve through reinforcement in response to disadvantages arising from mismating or hybridization [68, 69]. Reinforcement here is considered in its broad sense, i.e. “the evolution of mechanisms that prevent interbreeding between newly interacting incipient species, as a result of selection against inter-specific matings” [68]. Reinforcement is strongest when species co-evolve in sympatry where it typically results in pre-zygotic isolation evolving more rapidly than post-zygotic isolation [70, 71]. Here, post-mating reproductive isolation appears to be complete (inviability of hybrid eggs) yet pre-mating reproductive isolation is only partial suggesting that reinforcement between *Amblyomma* species in Mozambique has been weak. This pattern of reproductive isolation reflects a history of infrequent between-species contact [70, 71]. However, the degree of pre-mating reproductive isolation can vary spatially in response to differential competitive and sexual selection pressures [72, 73]. In Zimbabwe, tick dispersal events and between-species contacts are expected to be more frequent than in Mozambique due to higher cattle densities and more frequent host movements. It would therefore be interesting to test if female choosiness in mate choice is evolving more rapidly in Zimbabwe than in Mozambique, especially since augmentations in female choosiness could profoundly affect the future distributions of these ticks.

## Conclusion

We report the first field observations of hetero-specific mating between *A. variegatum* and *A. hebraeum* from sympatric areas in Mozambique. These results, coupled with previous experimental evidence of egg inviability resulting from hybridisation, suggest that sexual competition between these species provides a key mechanism that can, at least partially, explain the spatial segregation observed in Mozambique and Zimbabwe. Sympatry within the contact zone is relatively rare, which corresponds perfectly with the transient dynamics of sympatry predicted by theoretical sexual competition models. The extent to which environmental factors determine the location of the parapatric boundary is currently unknown. Further field, laboratory and modelling work is required to quantify the extent to which sexual competition displaces the parapatric boundaries away from any potential environmentally determined lines of equi-fitness between these two species.

## Additional file

Additional file 1:
**Supplementary information.** (DOCX 287 kb)

## Footnotes

1 In set notation, [*a*,*b*) = {*x*∈ℝ; *a* ≤ *x*<*b*}.
